# Exploring the Combination of Microgels and Nanostructured Fluids for the Cleaning of Works of Art

**DOI:** 10.3390/gels11060382

**Published:** 2025-05-23

**Authors:** Jacopo Vialetto, David Chelazzi, Marco Laurati, Giovanna Poggi

**Affiliations:** Chemistry Department & CSGI, University of Florence, 50019 Sesto Fiorentino, FI, Italy; jacopo.vialetto@unifi.it (J.V.); david.chelazzi@unifi.it (D.C.); marco.laurati@unifi.it (M.L.)

**Keywords:** microgels, microemulsions, nanostructured fluids, cleaning, cultural heritage

## Abstract

Cultural Heritage is a vital socioeconomic driver that must contend with works of art continuously exposed to degradation processes, which are further exacerbated by climate change. Aged coatings, varnishes, and soil can compromise the appearance of artworks, preventing their preservation and valorization. In response, soft matter and colloidal systems, such as nanostructured cleaning fluids (NCFs), have proved to be valuable solutions for safely and effectively cleaning works of art. Here, a novel cleaning system is proposed, for the first time employing microgels of poly(*N*-isopropylacrylamide) (PNIPAM) with surface chains of oligoethylene glycol methyl ether methacrylate (OEGMA) to favor shear deformation by lubrication. These microgels are loaded with NCFs featuring “green” solvents and different kinds of bio-derived or petroleum-based surfactants (non-ionic, zwitterionic). Rheological characterization of the combined systems highlighted a sharp transition from solid to liquid-like state in the 21–24 °C range when the zwitterionic surfactant dodecyldimethylamine oxide was used; the system displays a solid-like behavior at rest but flows easily at intermediate strains. At slightly higher temperature (>24 °C), an inversion of the G′, G″ values was observed, leading to a system that behaves as a liquid. Such control of rheological behavior is significant for feasible and complete removal of soiled polymer coatings from textured ceramic surfaces, which are difficult to clean with conventional gels, without leaving residues. These results position the PNIPAM-OEGMA microgels as promising cleaning materials for the conservation of Cultural Heritage, with possible applications also in fields where gelled systems are of interest (pharmaceutics, cosmetics, detergency, etc.).

## 1. Introduction

The conservation of Cultural Heritage continuously calls for new, advanced materials to be developed, characterized, and assessed, facing the growing issues of climate change that enhances degradation processes and the loss of valuable assets [1,2]. In particular, the cleaning of works of art represents a recurring task and a constant challenge [3] since soil, degradation products, and even vandalism [4] or wrong interventions, all cause the formation of unwanted additional surface layers that jeopardize the artworks. Therefore, cleaning systems must be designed to show high removal efficacy, while also being selective to the target layers, without inducing alteration of the original artistic/historical substrates. Over the last decades, this effort has pushed the development of soft matter and colloidal systems such as micelles, microemulsions, and gels, tailored to the removal of different kinds of unwanted layers from murals, canvas paintings, and other types of artworks [5]. In addition to providing tools to efficiently preserve art, research in this field is also proving as a drive to innovation that can potentially serve numerous linked applicative sectors, ranging from the detergency industry to cosmetics, pharmaceutics, and tissue engineering [6,7,8,9]. In this sense, gelled systems constitute one of the classes of materials with highest impact and versatility [9,10]. In particular, while different types of bulk hydro- and organogels are being developed [11,12,13,14] to preserve Cultural Heritage, colloidal microgel particles are still largely unexplored in the cleaning of works of art, despite representing a class of materials with utility in multiple scientific and technological fields [6,7,8,9,15,16].

Microgels are three-dimensional macromolecular polymer networks swollen by solvents, with size ranging from 100 nm to the micron range. They have dynamic, deformable, and permeable networks capable of responsivity to multiple stimuli depending on the constituting polymers (e.g., temperature, pH, or the presence of specific molecules), which cause changes in their diameter, mesh size, stiffness, or surface properties, making them ideal soft materials for a variety of applications [6]. For example, they can be used in compartmentalization and encapsulation of active species in a broad set of biological and biomedical applications [15,17], spanning from drug-delivery to wound-dressing and tissue replacements [18]. Alternatively, they find uses as rheology modifiers [19,20,21,22] and additives in inks for 3D printing [23,24], or for the fabrication of responsive coatings of surfaces [25,26] and fluid interfaces [27], exploiting their soft nature to tune their conformation on the target substrate and their in situ responsivity to external stimuli [28,29]. Such versatility makes microgels a key topic that can benefit from interdisciplinary research to develop new technologies [30]. In the framework of art conservation science, microgels are still in their infancy. Mazzuca et al. proposed gellan-based microgels for the cleaning of paper, and potentially also canvas and wood, evaluating the effects of temperature, polymer concentration, and methacrylation, as well as the presence of salts, on the viscosity and viscoelastic moduli of the gelled systems [31,32,33]. Rheological evidence was provided of a complex two-step aggregation mechanism of gellan in the microgels, with a double-yielding behavior exhibited in flow curves, and the gels were used to remove aged linseed oil and pressure adhesive tapes residues from paper substrates [31]. These studies show the potential of microgels in Cultural Heritage preservation, yet there are large gaps that need to be filled to further validate these systems and expand their application perspectives, especially for the removal of polymeric coatings from artistic surfaces, where the use of neat water is not effective.

A crucial aspect of this study is the combination of microgels with nanostructured cleaning fluids (NCFs), such as micelles and microemulsions, which is a novelty in art preservation and the main object of our contribution. NCFs are advantageous liquids for removing soil, aged coatings, and vandalism/overpaint, since they operate through different mechanisms than simple solvent blends, e.g., favoring the dewetting or selective swelling/detachment of unwanted layers from works of art [5]. While the confinement of aqueous NCFs in hydrogels has already been proposed [5,10,34,35], the combination of these fluids with microgels is, to the best of our knowledge, unprecedented in art cleaning. Furthermore, the effect of the interaction of micellar solutions and microemulsions with microgels is largely overlooked [36]. With respect to bulk hydrogels, the use of microgels as additives allows producing cleaning agents that have better adhesion and penetration on rough, highly textured substrates, and require less material for coating the targeted surface. Additionally, soft microgel particles flatten out when in contact with a solid surface, resulting in improved coverage of the underlying material.

Here, poly (*N*-isopropylacrylamide) (PNIPAM) microgels, containing surface chains of oligo (ethylene glycol) methyl ether methacrylate (OEGMA), were synthesized by surfactant-free semi-batch radical precipitation polymerization, and mixed with NCFs featuring “green” solvents and different kinds of surfactants (non-ionic, zwitterionic). PNIPAM is chosen as it is a benchmark, commonly investigated polymer for microgels due to the ease of synthesis and tunable responsivity [6,37]. Of particular interest is their switch from swollen, hydrated state at room temperature, to a collapsed state above the volume phase transition temperature (VPTT) of the polymer, approx. 32 °C for PNIPAM. The incorporation of ethylene glycol chains at the microgels’ surface is expected to favor shear deformation by lubrication [19], which could give applicative advantage to use the system on textured artistic surfaces. Diethyl carbonate (DEC) was selected as a “green” solvent to use in the fluids, since it is a promising solvent belonging to the alkyl carbonate class, with good solving power and low ecotoxicological impact. Either benzyl alcohol or cyclohexanol was added as a secondary organic solvent to further enhance the systems’ cleaning power. Different bio-derived or petroleum-based surfactants were used to stabilize the solvents’ nanosized droplets in the fluids’ continuous aqueous phase, to compare sustainable and benchmarks amphiphiles with applicative use in art cleaning. Understanding the mutual effect of the NCFs and microgels components on the rheology of these systems was also a key objective of this study, since it can help develop applications of these combined soft matter materials in different fields. Finally, the microgels loaded with the NCFs were used to remove a polymeric coating from a non-water sensitive clay surface representative of cleaning case studies in art preservation. Overall, this study aimed at expanding the palette of gelled materials available in Cultural Heritage conservation, exploring for the first time novel microgel-based cleaning systems that bring new emphasis on this class of soft matter materials previously mainly considered as coatings [38].

## 2. Results and Discussion

**Figure 1:**
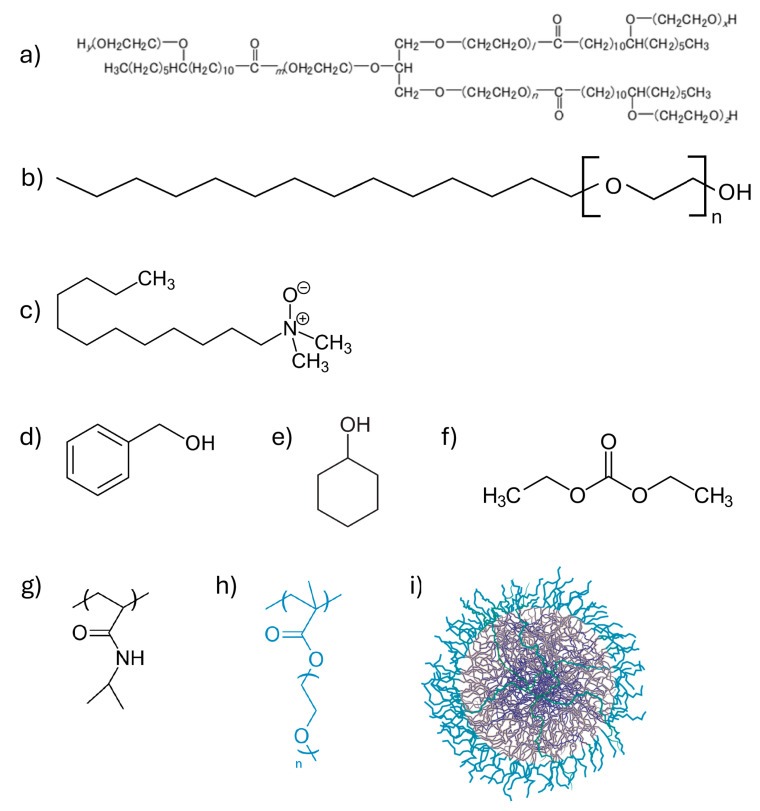
Structures of the main component of NCFs (**a**–**f**) and microgels (**g**,**h**). (**a**–**c**) NI1, NI2 and ZW, respectively; (**d**) BA; (**e**) CH; (**f**) DEC; (**g**) NIPAM; (**h**) OEGMA (M_n_ = 2000); (**i**) microgel structure.

In this study, we selected three NCFs that share the same dispersed phase, i.e., diethyl carbonate, which is immiscible with water and is present in comparable amounts across all the formulations. The main difference between these systems lies in the surfactant used for the preparation of the NCFs: two are non-ionic, and one is zwitterionic (see Figure 1). Based on the surfactant type, different cosolvents, each partially soluble in water, were incorporated. The aim was to investigate the interaction between these cleaning systems and a previously characterized microgel, employed here for the first time as a thickening agent, within a nanostructured liquid phase. The microgels used are made using a semi-batch precipitation polymerization protocol with PNIPAM as the main monomer, N, N′-methylenebis (acrylamide) (BIS, 5 mol %) as crosslinker, and present OEGMA chains (M_n_: 2000) on their surface. OEGMA chains allow modulating their rheological properties as well as increasing their biocompatibility [19]. Details on the synthesis protocol and microgel characterization are reported in Materials and Methods and Supporting Information (Figures S1 and S2). The ultimate objective was to combine the cleaning efficiency of a micelle-based system with a high-surface-area thickener for the cleaning of artistic artifacts. Prior to this, we conducted a preliminary assessment of the physicochemical properties of the individual components, as well as of the complete nanostructured fluid in the presence of the microgel.

The size of micelles in non-thickened micellar solutions, obtained using different surfactants, is reported in Table 1, in terms of hydrodynamic diameter (D_h_), and it is in line with the information already available in the literature, for the same or similar surfactants [39,40,41]. As expected, non-ionic surfactants polyethylene glycol-40 hydrogenated castor oil (NI1) and polyoxyethylene alkyl (12–14) ether (12 ethylene oxide) (NI2), having more bulky structures, lead to the formation of larger nanostructures than dodecyldimethylamine oxide (ZW). When cosolvents (cyclohexanol, CH or benzyl alcohol, BA) are added to micellar solution, the size of the aggregates is maintained for NI2, and only slightly affected when NI1 and ZW are present. The successive addition of the water insoluble dispersed phase (diethyl carbonate, DEC) leads to the expected formation of larger micelles that expand to allow for DEC inclusion. This is especially evident for NCF2 (i.e., nanostructured cleaning fluid stabilized with NI2 surfactant), and less so for NCF1. We then mixed the obtained NCFs with the microgels at a concentration of 0.001 wt%. At this dilute microgel concentration the DLS correlogram (an example of which is reported in Figure S3) is characteristic of a single size distribution, which, due to the resulting small size, we attribute to the micelle dimension in suspension. DLS data show (Table 1) that both surfactant micelles and the complete microemulsions containing DEC remain stable after mixing with the microgels, and that their size does not change significantly. This occurs regardless of the main surfactant or cosolvent used for the stabilization of the organic phase. We note that increasing the microgel concentration results in multiple scattering in the DLS measurements and in the appearance of multiple secondary peaks with low intensity in the size distribution, which we attributed to the presence of aggregates in suspension and preclude a detailed characterization of the microgel’s size with this technique.

Optical microscopy images of microgels and nanostructured cleaning fluids (µNCFs) provided qualitative insights on the overall sample appearance and stability after mixing at a microgel concentration two orders of magnitude higher than DLS experiments, and closer to the concentration range of interest for our target application.

**Figure 2:**
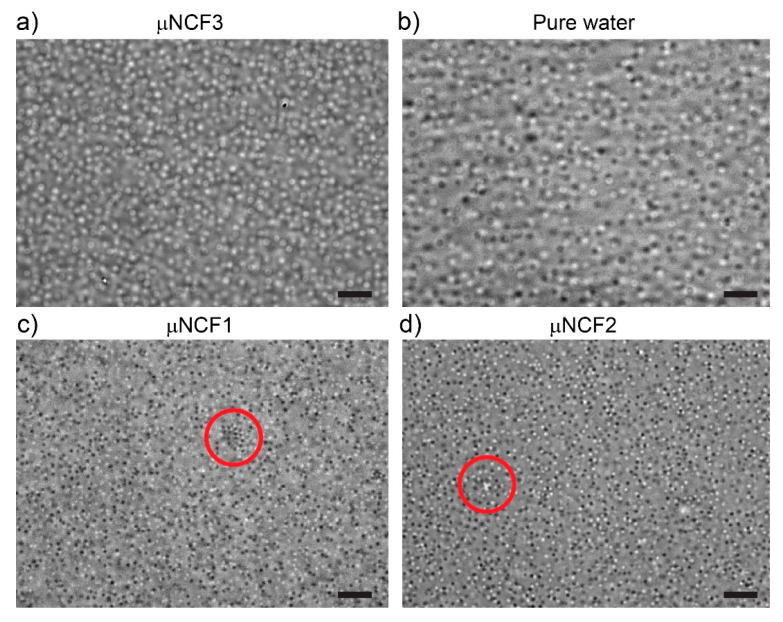
Mixtures of microgels and nanostructured cleaning fluids (µNCFs). Optical microscopy images of (**a**) µNCF3; (**b**) blank (microgels in pure water); (**c**) µNCF1; (**d**) µNCF2. The red circles in (**c**,**d**) highlight cluster of aggregated microgels. Microgel concentration: 0.125 wt%. Scale bars: 5 μm.

A visual comparison between the images in Figure 2 indicates that the microgels remain well dispersed in NCF3 (Figure 2a), where they maintain a similar size as in the control experiment in pure water (Figure 2b). This suggests that the microgels are in a good solvent in NCF3 as indicated by a significant network swelling, similar to the pure water case at the same temperature (20 ± 2 °C). Instead, in both NCF1 and NCF2 (Figure 2c,d), the microgels deswell appreciably, and a few clusters of aggregated particles are visible throughout the sample (marked with red circles in the figures).

Deswelling hints that homogeneous mixing of microgels and NCF1 and NCF2 at higher concentrations, as required for their use as thickening agents, might not be possible due to partial aggregation and phase separation of the microgels within the sample. Indeed, the mixture of microgels at a concentration >1 wt% with NCF1 and NCF2 resulted in complete phase separation of the polymeric material from the microemulsion suspension. Instead, the microgels + NCF3 system (µNCF3) remained stable for a long period of time (min 30 days).

**Figure 3:**
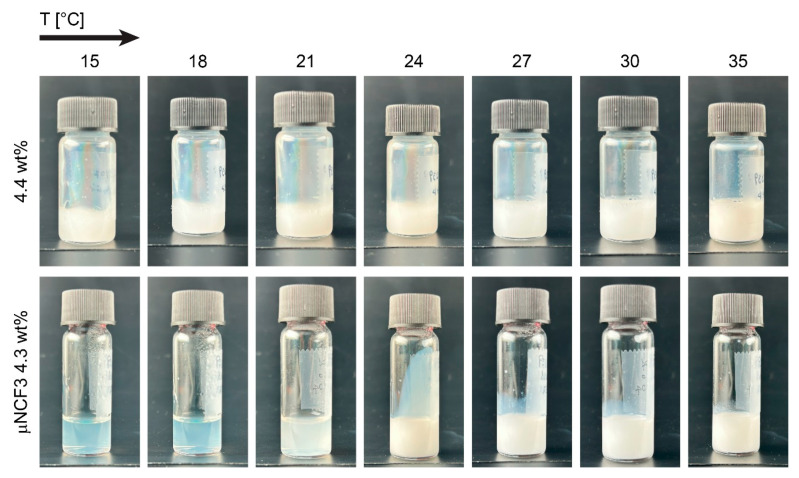
Temperature-responsive suspensions. Images of vials containing dense suspensions of microgels in water at 4.4 wt% (**top**) and µNCF3 at 4.3 wt% (**bottom**), at increasing temperatures, as indicated. Vial size: 16 mm (**top**), 14 mm (**bottom**).

Having in mind the application of the combined systems for cleaning operations on artistic surfaces, we prepared microgel suspensions in water at an effective volume fraction of φ = 1.07, corresponding to 4.4 wt% (see Section 4), with comparable NCF3 wt%. Such an effective volume fraction in water is chosen as it yields a solid-like paste at room temperature. The µNCF3 system maintains a solid-like state, indicating that the microgels at this concentration disperse well in the microemulsion and act as efficient thickening agents. Quite surprisingly, at 15 °C, the µNCF3 system appears more transparent, i.e., it scatters less incident white light than the sample in water (Figure 3, left), presumably due to decreased discrepancy between the refractive index of the suspension and that of the particles [42].

Considering the well-known thermal responsivity of pNIPAM microgels in water, we looked at the behavior of the dense microgel suspensions upon increasing temperature, starting with visual inspection (Figure 3). The suspension in pure water does not show any significant optical variation across the microgel’s volume phase transition temperature (VPTT), which is at approximately 31–32 °C for our particles (see Figure S2), with only a slight increase in opalescence at high temperature [43]. Conversely, the sample in NCF3 exhibits a peculiar behavior upon temperature increase, with transition from a clear, almost transparent, state to an opalescent one at high temperature. This transition is accompanied by a dramatic increase in sample fluidization (see below) and can therefore be attributed to a significant decrease in the effective volume fraction of the suspension due to the shrinkage of the microgels. This shrinkage by solvent expulsion from the polymer network increases the particle refractive index and scattering of the incoming white light, thereby causing the transition to an opalescent state [43].

**Figure 4:**
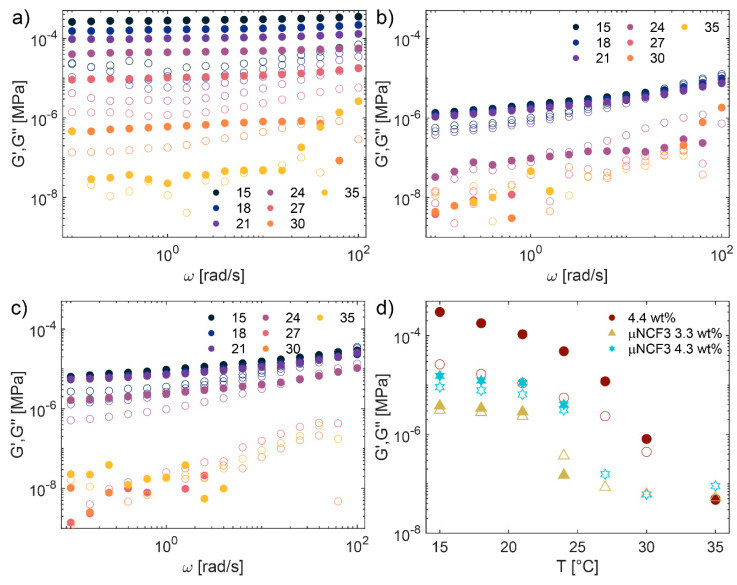
Linear rheology of microgels in water and of µNCF3 systems. (**a**–**c**) Dynamic frequency sweeps at γ_0_ = 0.5% for different temperatures as indicated. Solid symbols represent the storage moduli (G′) and open symbols the viscous moduli (G″). (**a**) Microgels in water at 4.4 wt%. (**b**) µNCF3 system at 3.3 wt%. (**c**) µNCF3 systems at 4.3 wt%. (**d**) Storage and viscous moduli at ω = 10 rad/s as a function of temperature for all systems.

**Figure 5:**
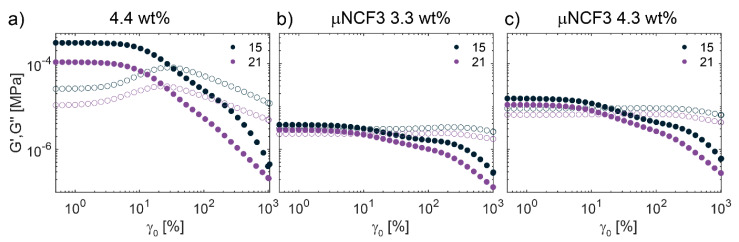
LAOS of microgels in water and of µNCF3 systems. Storage (*G*′, solid symbols) and loss (*G*″, open symbols) moduli from strain amplitude sweeps at 15 °C (black) and 21 °C (violet), and *ω* = 10 rad/s. (**a**) Microgels in water at 4.4 wt%. (**b**) µNCF3 at 3.3 wt%. (**c**) µNCF3 at 4.3 wt%.

For quantitative characterization of the samples’ viscoelastic behavior, we performed rheological measurements in the presence and absence of NCF3. Figure 4a–c shows the frequency-dependent linear storage (G′) and loss (G″) moduli as a function of frequency for the microgel suspension in water at 4.4 wt% (Figure 5a), in NCF3 at 3.3 wt% (Figure 4b), and in NCF3 at 4.3 wt% (Figure 4c), in the 15–35 °C range. For the sample in water, G′ is always higher than G″, and the response is predominantly solid-like. We note that, at this effective volume fraction, the sample is well within the jamming limit as estimated by Pellet and Cloitre [44]; therefore, the microgels forming the solid network are highly deformed, compressed, and possibly interpenetrated [20]. Both moduli decrease with temperature, and at the highest investigated temperature (35 °C) G′ ≃ G″, indicating transition to a more liquid-like state. Below this temperature, no crossover between the moduli is observed in the probed range of frequencies: the microgels do not show significant diffusion or structural relaxation over this timescale. On the other hand, the µNCF3 system shows a frequency-dependent behavior at low temperatures, with both moduli increasing at higher ω. In particular, the sample is solid-like (G′ > G″) up to ω ≃ 10 rad/s, while the loss modulus becomes higher than the storage modulus at higher frequencies. With respect to the sample in water, both G′ and G″ are only weekly dependent on temperature up to 21 °C. After this threshold, we instead observe a sharp transition from a solid-like to liquid-like behavior, indicated by pronounced decrease in G′ and G″ values. This transition is concentration-dependent, happening above 21 °C at 3.3 wt%, and above 24 °C at 4.3 wt%, similar to what is observed for microgels dispersed in pure water, for which a system at a lower effective concentration is closer to the liquid phase and therefore a smaller variation in particle size induced by temperature is enough to shift the effective volume fraction below the liquid transition.

These results are visually summarized in Figure 4d, where we plot the storage and viscous moduli at ω = 10 rad/s as a function of temperature for the investigated samples. As already observed, G′ is always higher than G″ for the sample in water, with both moduli decreasing as a function of temperature, and overlapping at 35 °C. G′ and G″ of µNCF3 systems are significantly lower than in pure water, and increase with the microgel concentration. While they remain approximately stable at low temperatures, they show a significant decrease above 21–24 °C, with G′ becoming lower than the detection limit, indicating a sharp transition from solid to liquid-like state. We anticipate that this behavior is of great interest in light of their usage for the cleaning of artworks, indicating that the samples can be applied at T < 21 °C as a paste on the target surface, and subsequently rapidly wiped out with the help of a gentle temperature increase above 24 °C, which most artistic surfaces can withstand. Alternatively, at a temperature lower than 24 °C, an increase in the stress applied to the systems can lead to its fluidification, favoring its easy and gentle removal, as also discussed below.

We then performed large-amplitude oscillatory sweep (LAOS) experiments. In Figure 5, we plot the first-harmonic responses in strain amplitude sweeps at *ω* = 10 rad/s at 15 and 21 °C. At low strains, the response is in the linear regime, G′ is higher than G″ and both moduli are approximately constant. At larger strains the response becomes non-linear, with G′ that decreases monotonically, while the G″ response is sample-dependent. For the microgel suspension in water (Figure 5a), we recover the weak strain overshoot in G″ commonly observed in a variety of soft glassy materials, including dense microgel suspensions [45,46,47,48], which is associated with continuous transition from recoverable to unrecoverable deformation [49]. The G′, G″ crossover, considered a rough estimation of the material’s yield point, occurs at γ_0_ ≈ 30% or γ_0_ ≈ 25% at 15 °C and 21 °C, respectively. Above this value, G″ > G′, and the response is primarily viscous-like. µNCF3 systems, on the other hand, do not show significant variation in G″ at intermediate and high strain. This behavior, named strain thinning, is typically observed for polymer solutions and melts, and indicates that the cages forming the entire network flow very easily at intermediate and large applied strain amplitudes. Such a sample response could be attributed to a lower effective volume fraction of the microgels dispersed in the NCF3 solution due to a slight decrease in particle size, which is responsible also for the lower moduli observed in the frequency sweeps experiments (Figure 4). A lower effective volume fraction yields samples with less compressed particles and more flexible cages. The G′, G″ crossover is observed at γ ≈ 10% (15 °C) or γ ≈ 8% (21 °C) for µNCF3 at 3.3 wt%, γ ≈ 20% (15 °C) or γ ≈ 15% (21 °C) for µNCF3 at 4.3 wt%. Therefore, yielding happens at lower γ values for the µNCF3, and the yielding point can be tuned by varying the microgel concentration. Overall, these results indicate that the µNCF system displays solid-like behavior at rest, while it flows easily at intermediate applied strains.

**Figure 6:**
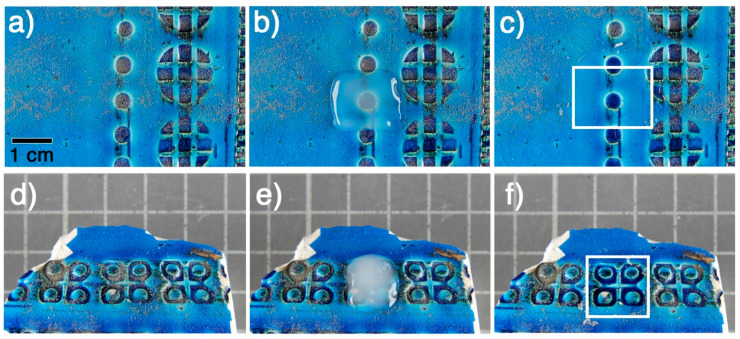
Application of the µNCF3 system at 4.3 wt% on a clay sample coated with a soiled Plextol B500^®^ layer. All the images have the same magnification. One square on the background measures 1 cm^2^, as clearly indicated by the scale bar in (**a**). (**a**,**d**) Soiled areas before application. (**b**,**e**) The same area during application. (**c**,**f**) After application, the swollen polymeric coating is easily removed using a humid cotton swab. The white squares in the images are added to highlight the clean areas.

To assess the cleaning efficacy of the µNCF3 system, a series of tests were performed to remove a soiled acrylic coating, Plextol B500^®^, from a blue glazed clay of artistic significance, belonging to the well-known collection *Rimini Blu* (see Figure 6). For these tests, a small amount of µNCF3 (microgel concentration 4.3 wt%) was applied to an area of approximately 1.5–2 cm^2^ on the fragment and left in place for 20 min. The treated area was covered with a plastic film to prevent evaporation during application. After the treatment period, the gel was removed using a spatula. In a second test, illustrated in Figure 6d–f, the cleaning system µNCF3 was removed with a slightly heated spatula, allowing the fluidified gel to be absorbed with a cotton disk, as a result of the changes in the viscoelastic properties of microgels loaded with the microemulsion upon heating (see Figure 4d). It is worth noting that Figure 6e was taken during the heating of µNCF3, which was already starting to become less transparent, as it occurs upon temperature increase (see Figure 3). Nevertheless, after the application and removal of the cleaning system µNCF3, the Plextol B500^®^ layer, which was swollen by the action of the cleaning fluid, was eliminated by gently wiping the surface with a cotton swab slightly moistened with demineralized water. Cleaned areas are highlighted by white squares in Figure 6c,f.

**Figure 7:**
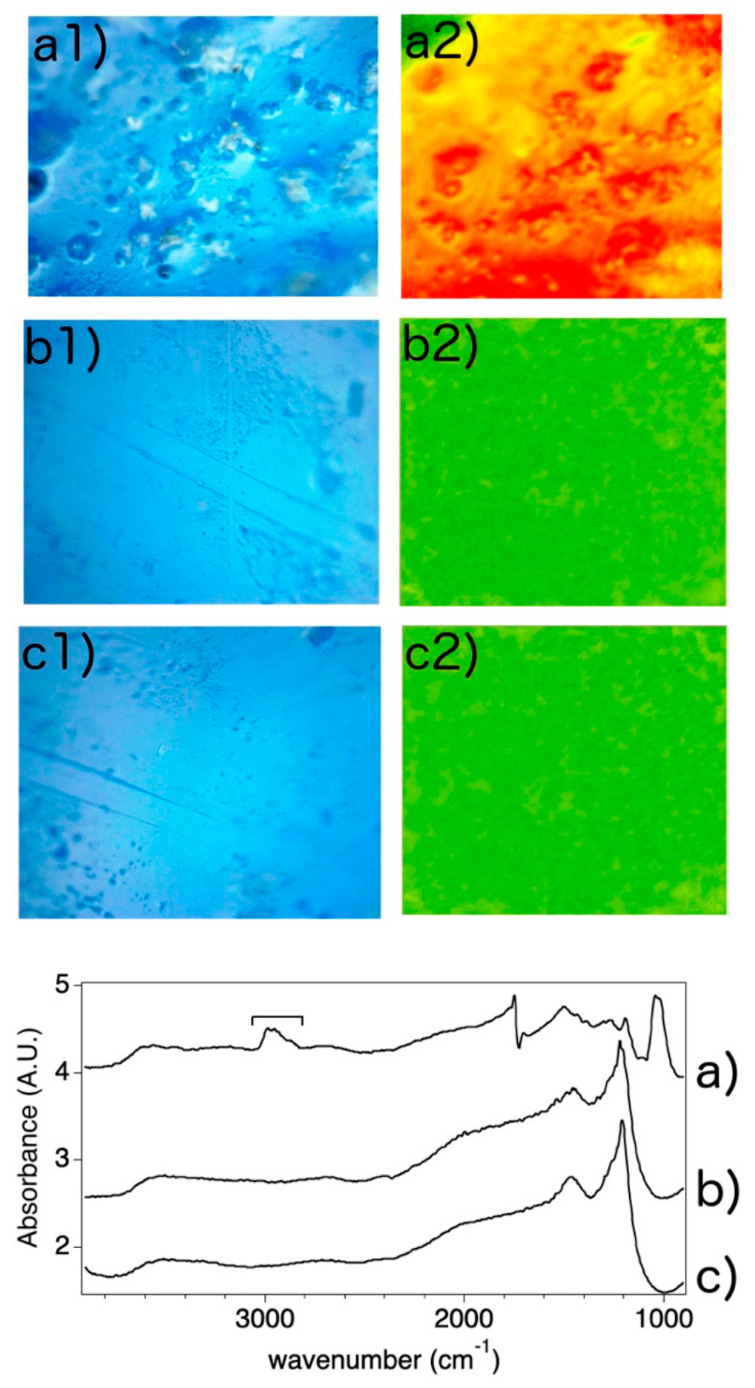
Visible light (**a1**–**c1**) and 2D false colors IR maps (**a2**–**c2**) of a clay sample: (**a**) coated with a soiled Plextol B500^®^ layer; (**b**) after the removal of the coating using the µNCF3 system; (**c**) pristine surface reference (uncoated). The visible images are 500 × 500 µm^2^, the IR maps are 700 × 700 µm^2^. The maps were obtained by converting to false colors the absorbance in the 3080–2820 cm^−1^ region of the reflectance IR spectra acquired from the sample’s surface. Yellow–red pixels in the maps correspond to spectra that show absorptions in the region, due to CH stretching bands of the acrylate polymer chains in the coating (spectrum a in bottom panel). Green pixels indicate no absorption in the pristine and cleaned sample areas (spectra b and c).

FTIR 2D imaging confirmed homogeneous removal of the soiled Plextol B500^®^ at the micro-scale from the glazed clay surface by the µNCF3 system. Figure 7 shows the 2D false color IR maps imaging the CH stretching region from 2820 to 3080 cm^−1^ on the tested clay sample. When the glazed surface is coated with Plextol B500^®^, strong absorption is detected, as shown by the yellow–red colored pixels in the map (Figure 7(a2)). After the application of microgels loaded with the microemulsion, no absorption in the same CH stretching region is detected from the surface (as shown by the green color in Figure 7(b2)), as for the pristine glazed surface that was not coated with the polymer (Figure 7(c2)). The spectra collected on the surface after cleaning perfectly match those from the pristine surface. Notably, the absence of absorptions in the CH stretching region is also an indication that no residues from the microgel’s suspension were left on the cleaned surface. It is worth mentioning that the detection limit of the FPA detector for polymeric substances (e.g., polyvinyl alcohol) with this setup was found to be ca. 0.02 pg μm^−2^ [50]. This confirms that satisfactory and residue-less removal of the polymer coating was achieved down to the micro-scale by the application of the µNCF3 system.

## 3. Conclusions

Overall, we explored the possibility of loading PNIPAM-based microgels with nanostructured cleaning fluids, combining the known cleaning potential of these fluids with the rheological properties of microgel suspensions. To achieve this, we selected three cleaning fluids having the same dispersed phase (i.e., DEC). The systems differ in the chemical nature of the surfactants and cosolvent added to each nanostructured fluid. As expected, the hydrodynamic diameter of micelles obtained using different surfactants are quite different, spanning from 5 to 18 nm. The addition of cosolvents and of DEC leads to an increase in the size of the aggregates. Interestingly, when the microgels are added at low concentration, the average size of the aggregates in all the NCFs does not change significantly. With increasing microgel concentration in both NCF1 and NCF2, the microgels deswell appreciably, and a few clusters of aggregated particles are visible, while swelling is observed in NCF3, as well as in pure water. At about 4% wt microgel content, significant phase separation occurred in µNCF1 and µNCF2, while a stable, quite transparent dispersion was obtained for µNCF3. Therefore, rheological tests were performed only on the µNCF3 system, showing that when the nanostructured fluid is added to microgels, G′ and G″ decrease. More interestingly, µNCF3 shows a particular behavior upon temperature increase, passing from solid to liquid state above 24 °C. LAOS experiments elucidated another interesting feature of the µNCF3 system, which, differently from microgels in pure water, behaves as a solid at rest and flows easily at intermediate applied strains. The cleaning tests confirmed that the system effectively swells an acrylic polymer coating, which could then be easily removed from glazed clay with gentle mechanical action. Two different methods, using either mild temperature or moderate strain, were employed to remove the gel from the ceramic surface, taking advantage of the specific properties of the combined system. 2D FTIR imaging verified the complete removal of the soiled polymeric coating, with no detectable residues remaining on the sample surface—either from non-volatile components of the cleaning fluid (such as the surfactant) or from the polymeric microgel. Overall, the combined system proposed in this study broadens the range of advanced soft matter cleaning technologies available for the conservation of artistic surfaces, offering an additional, effective tool for professionals dedicated to the preservation and of Cultural Heritage and its transmission for future generations.

## 4. Materials and Methods

### 4.1. Reagents

Polyethylene glycol-40 hydrogenated castor oil surfactant (NI1, Nikko Chemicals, Tokyo, Japan), polyoxyethylene alkyl (12–14) ether (12 ethylene oxide) surfactant (NI2, Nikko Chemicals, Tokyo, Japan), and dodecyldimethylamine oxide surfactant (ZW, Merck, Darmstadt, Germany, 30 wt% in H_2_O) were used without further purification. Cyclohexanol (CH), benzyl alcohol (BA), and diethyl carbonate (DEC) were acquired from Merck (Darmstadt, Germany) and used for nanostructured cleaning fluids preparation, without further purification (99.8%). Oligo (ethylene glycol) methyl ether methacrylate (OEGMA) (Merck, Darmstadt, Germany, 50 wt% in H_2_O, M_n_: 2000), N,N′-methylenebis (acrylamide) (BIS, Honeywell Fluka, Charlotte, NC, USA, 99.0%) and potassium persulfate (KPS, Sigma–Aldrich 99.0%, Burlington, MA, USA) were used without further purification. *N*-isopropylacrylamide (NIPAM, TCI Europe, Zwijndrecht, Belgium, 98.0%) was purified by recrystallization in 40/60 *v*/*v* toluene/hexane [28]. Water used for all the experiments was purified by a Millipore system (resistivity > 18 MΩ·cm).

### 4.2. Systems Preparation

*Microgel Synthesis*. Microgels containing OEGMA surface chains were synthesized by surfactant-free semi-batch radical precipitation polymerization already published elsewhere [19]. Briefly, NIPAM (3 g) and BIS (1 mol %) were dissolved in 350 mL of MilliQ water and purged with nitrogen for 1 h at 70 °C. Separately, OEGMA (4.9 mol %) in 20 mL of MilliQ water and KPS (50 mg) in 10 mL of MilliQ water were purged with nitrogen for 1 h. The reaction started by adding KPS to the reaction flask with NIPAM and BIS. After 20 min, the OEGMA monomer solution was injected into the flask at a rate of 0.33 mL/min. The reaction was carried out for a total time of 5 h and then quenched by opening the flask and placing it in an ice bath. The resulting suspension was dialyzed for a week, purified by 6 centrifugation cycles and resuspension of the sedimented particles in pure water, and freeze-dried. The resulting particle hydrodynamic diameter in water at 25 °C is 1500 ± 70 nm.

*Nanostructured Cleaning Fluids Preparation*. In Table 2, the composition of the three selected nanostructured cleaning fluids, i.e., NCF1, NCF2, and NCF3 is reported. As a general procedure, surfactants were mixed with water and allowed to solubilize, which, in the case of NI1 required about 24 h of stirring at room temperature, while it was quite fast (few minutes) for the other two surfactants. Once a transparent system is obtained, cosolvent is added to the mixture, gradually, allowing the systems to reach the equilibrium before proceeding to the next step. Finally, DEC is added to each mixture, dropwise, until a stable, transparent system is obtained.

*Combination of Microgel and NCFs*. For the preparation of combined systems, a fixed amount of freeze-dried microgel was added to an aliquot of each NCF, until the desired concentration of solid is reached. Depending on the type of measurements, concentrations of microgels spanning from 0.001 wt% to 4.4 wt% were prepared. After preparation, the systems were mixed using an orbital shaker for about 24 h. The combined systems are therefore labelled as µNCF1, µNCF2, and µNCF3, depending on the nanostructured fluid used for the preparation.

### 4.3. Experimental Methods

*DLS*. Dynamic light scattering (DLS) experiments were performed using a Zetasizer PRO Red Label (Malvern, UK) in back-scattering mode. The temperature was set at 25 °C and the quartz cells let to equilibrate for 8 min prior to performing four consecutive measurements of 20 runs of 20 s each. For DLS experiments as a function of temperature, the temperature was scanned from 19 to 51 °C with 2 °C steps. Each temperature was let to equilibrate for 10 min before four consecutive measurements of 15 runs each were performed.

*Optical Microscopy*. Optical microscopy images were taken in bright field contrast using an inverted Nikon Eclipse Ts2R microscope equipped with a 60× oil immersion objective (NA = 1.40) and a Hamamatsu ORCA-Flash 4.0 V3 CMOS camera. The suspension was let to equilibrate for 7 days prior to imaging at room temperature (25 ± 2 °C).

*Rheology*. The particle effective volume fraction *φ* in water was estimated as ϕ=k·wt%, where *k* is a constat determined by fitting the relative viscosity of dilute microgels suspensions (0.05–0.3 wt%) with respect to that of the pure solvent, to the Einstein–Batchelor relation $\eta_{rel}=1+2.5\phi +5.9\phi^{2}$. The resulting *k* for the microgels used in this work is 24.4 ± 0.3 (see Figure S1). Rheological measurements were performed using a Discovery HR-3 rheometer (TA Instruments, New Castle, DE, USA), with a cone-plate geometry (stainless steel, diameter: 40 mm, truncation gap: 54 um). A Peltier in contact with the lower plate ensured a constant temperature, and a solvent trap consisting of an enclosure with a solvent seal at the top and a wet tissue adhered to its interior was used to avoid evaporation. A rejuvenation protocol was implemented before each measurement to minimize variabilities due to sample loading and aging. We first applied an oscillatory shear for 120 s with a large strain amplitude (*γ* = 600%) at frequency *ω* = 1 rad/s, during which all samples showed a liquid-like behavior. We then applied a second oscillatory shear with a low-strain amplitude (*γ* = 0.5%) at frequency *ω* = 10 rad/s, until a steady state response in the viscoelastic moduli was reached. For all samples, such a steady state response was obtained within 120 s. Frequency sweep experiments were performed at *γ* = 0.5%, varying *ω* from 100 to 0.1 rad/s. Oscillatory shear experiments as amplitude sweeps were performed at constant frequency, with strain amplitude *γ*_0_ varying from 0.5% to 1000%.

*FTIR 2D Imaging*. Fourier Transform Infrared spectroscopy 2D Imaging was performed on the glazed clay surface (see “Cleaning tests” below) before and after the removal of a commercial acrylic coating in water at 10%, i.e., Plextol B500^®^ coating, by the microgel mixture with the NCF, as well as on pristine surface that had not been coated. Measurements were carried out using a Cary 620–670 FTIR microscope equipped with a Focal Plane Array (FPA) 128  ×  128 detector (Agilent Technologies, Santa Clara, CA, USA) and a 15× Cassegrain objective, in reflectance mode directly on the clay surface, with open aperture and a spectral resolution of 8 cm^−1^. A total of 128 scans were acquired for each spectrum. Background spectra were collected on a golden platelet. Each analysis produces a “tile” 2D map of 700  ×  700 μm^2^ (128  ×  128 pixels), where each pixel is 5.5  ×  5.5 μm^2^ and yields an independent IR spectrum. With this setup, the detection limit of the FPA detector for polymeric materials has been found to be approximately 0.02 pg μm^−2^ [50]. The spectra were analyzed using the Agilent Resolution Pro software (Agilent Technologies, version 5.4.1.3412). The false color 2D IR maps were obtained by imaging the main diagnostic bands of the acrylate polymer chains in the coating. In the 2D FTIR maps, the absorbance of the bands was converted to a chromatic scale as follows—green  <  yellow  <  red—where green indicates no absorptions, and red intense absorptions.

### 4.4. Cleaning Tests

The assessment of the most promising combination of nanostructured fluid and microgels was conducted on a blue glazed clay sample belonging to the iconic collection *Rimini Blu*, originally designed by Aldo Londi in 1959 during his collaboration with the Italian company Bitossi and still in production today. To that aim, the clay fragment was covered with Plextol B500^®^ (CTS, Altavilla Vicentina, Italy, acrylic resin in aqueous dispersion at 10% wt) mixed with artificial dirt [51], to mimic a soiled protective/adhesive layer that needs to be removed. For tests, a small amount of microgels with NCFs was placed on a small area (approx. 1.5–2.0 cm^2^) of the fragment for 20 min. The treated area was covered with a plastic foil (inert to the NCFs) to prevent evaporation during application. Thereafter, the gel was removed with a spatula and the swollen layer was gently removed using a cotton swab soaked in water. Pictures were taken before and after application. The assessment of the cleaning efficacy was also conducted using FTIR 2D imaging, as reported above.

## Figures and Tables

**Table 1:** Hydrodynamic diameter (D_h_) at 25 °C from dynamic light scattering (DLS). Microgel concentration: 0.001 wt%.

Sample	D_h_ [nm]	
	No Microgels	With Microgels
NI1	17.5 ± 0.1	18.1 ± 0.2
NI1 + CH	14.4 ± 0.1	14.1 ± 0.1
NCF1	26.8 ± 0.2	25.7 ± 0.2
NI2	7.0 ± 0.1	7.5 ± 0.1
NI2 + CH	7.9 ± 0.1	7.7 ± 0.1
NCF2	55.2 ± 2.1	57.6 ± 2.6
ZW	4.5 ± 0.1	4.7 ± 0.2
ZW + BA	10.9 ± 0.2	11.5 ± 0.2
NCF3	13.4 ± 0.1	13.4 ± 0.1

**Table 2:** Nanostructured cleaning fluids (NCFs) composition. The remaining percentage, not explicitly indicated, is that of water, the main component of the systems.

*Component*	*NCF1*	*NCF2*	*NCF3*
Surfactant	NI1 (4.7%)	NI2 (4.8%)	ZW (5.0%)
Dispersed phase	DEC (4.0%)	DEC (4.0%)	DEC (3.0%)
Cosolvent	CH (4.0%)	CH (4.0%)	BA (2.0%)

## Data Availability

The original contributions presented in this study are included in the article/Supplementary Materials. Further inquiries can be directed to the corresponding author.

## Supplementary Materials

The following supporting information can be downloaded at: https://www.mdpi.com/article/10.3390/gels11060382/s1, Figure S1: Experimental viscometry of dilute PNIPAM-OEGMA microgel suspensions in Milli-Q water at 20 °C. Figure S2: DLS as a function of temperature for dilute PNIPAM-OEGMA microgels dispersed in Milli-Q water; Figure S3: Correlogram obtained from DLS measurements on a suspension containing microgels (0.001 wt%) dispersed in NCF3.
