# Supplementary material for: Exploring the Combination of Microgels and Nanostructured Fluids for the Cleaning of Works of Art
Source: Gels. 2025 May 23;11(6):382. doi: 10.3390/gels11060382 (PMC12192406; doi:10.3390/gels11060382)
Supplement: Supplementary file 1 [file gels-11-00382-s001.zip › gels-3643809-supplementary.pdf]

Supplementary Materials

Exploring the Combination of Microgels and Nanostructured Fluids for the Cleaning of Works of Art

Jacopo Vialetto, David Chelazzi, Marco Laurati and Giovanna Poggi *

Chemistry Department & CSGI, University of Florence, 50019 Sesto Fiorentino, FI, Italy; jacopo.vialetto@unifi.it (J.V.); david.chelazzi@unifi.it (D.C.), marco.laurati@unifi.it (M.L.)

* Correspondence: giovanna.poggi@unifi.it

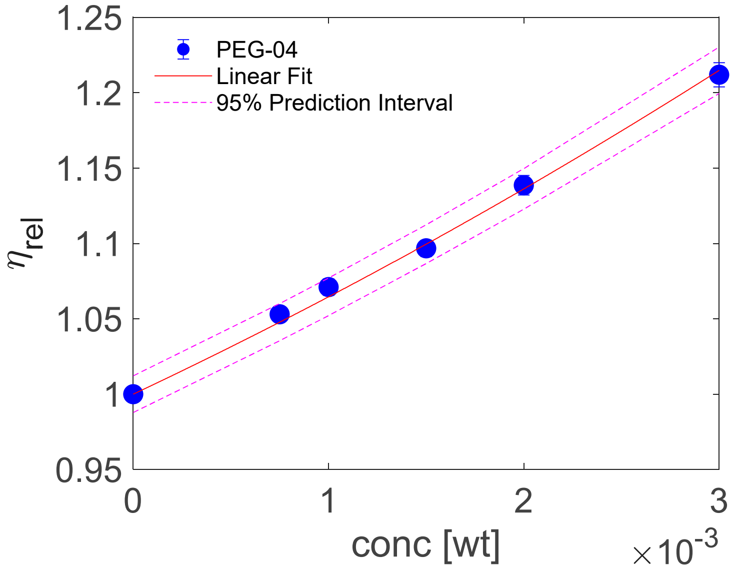

Figure S1. Experimental viscometry of dilute PNIPAM-OEGMA microgel suspensions in Milli-Q water at 20°C. Data are fitted with the Einstein Batchelor equation (redline). Pink dotted lines represent 95% prediction interval.

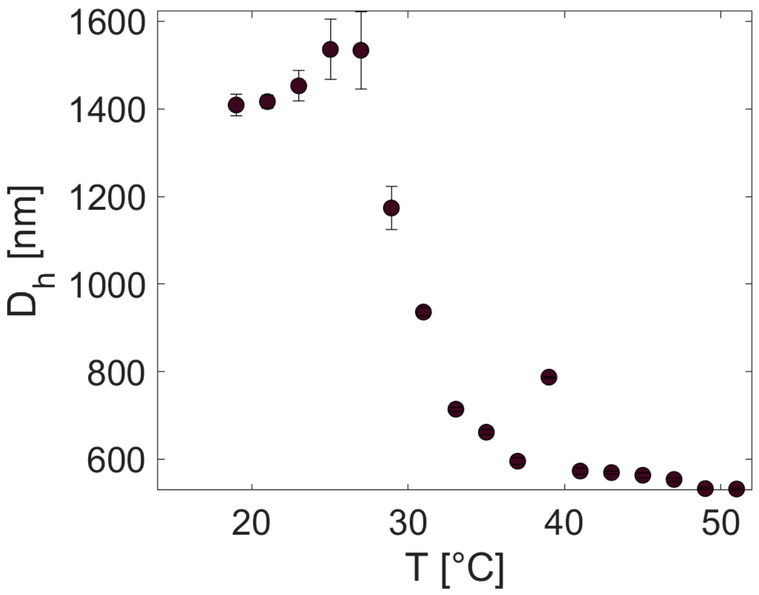

Figure S2. DLS as a function of temperature for dilute PNIPAM-OEGMA microgels dispersed in Milli-Q water.

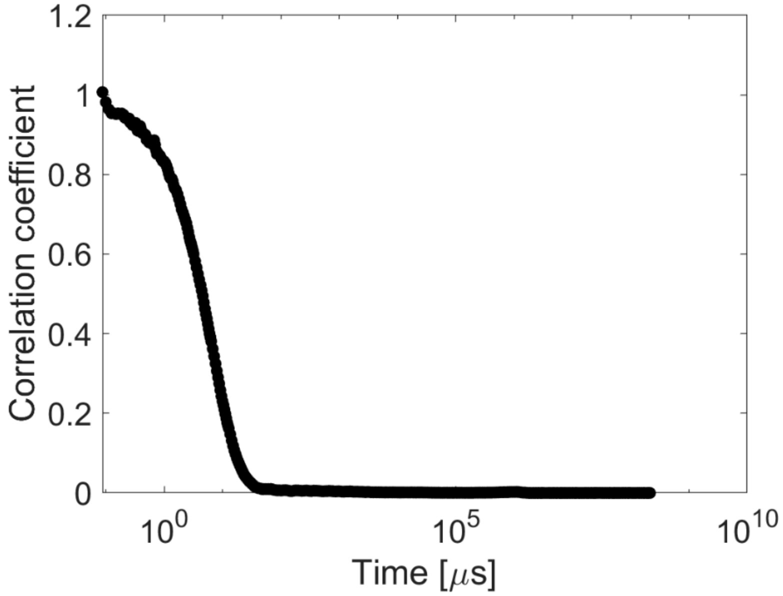

Figure S3. Correlogram obtained from DLS measurements on a suspension containing microgels (0.001 wt%) dispersed in NCF3.
